# Changes in Radiosensitivity to Gamma-Rays of Lymphocytes from Hyperthyroid Patients Treated with I-131

**DOI:** 10.3390/ijms231710156

**Published:** 2022-09-05

**Authors:** Valentina Dini, Massimo Salvatori, Mauro Belli, Maria Elena Lago, Alessandra Nosdeo, Donatella Pia Dambra, Luisa Lo Conte, Ilaria Pecchia, Alessandro Giordano

**Affiliations:** 1 National Center for Innovative Technologies in Public Health, Istituto Superiore di Sanità, 00161 Rome, Italy; 2 Istituto Nazionale di Fisica Nucleare (INFN), Sezione di Roma1, Department of Physics, University La Sapienza, 00185 Rome, Italy; 3 Nuclear Medicine Unit, Fondazione Policlinico Universitario A. Gemelli IRCCS, 00168 Rome, Italy; 4 Department of Radiological and Hematological Sciences, Università Cattolica del Sacro Cuore, 00168 Rome, Italy; 5 Nuclear Medicine Unit, ARNAS Garibaldi-P.O. Nesima, 95122 Catania, Italy; 6 Associazione Donatori Sangue “La Rete di Tutti”, 00145 Rome, Italy; 7 Nuclear Medicine Unit, “Vito Fazzi” Hospital, 73100 Lecce, Italy

**Keywords:** I-131 therapy, peripheral blood lymphocytes, micronucleus tests, H2AX histone phosphorylation, radio-sensitivity

## Abstract

This study investigated the peripheral blood lymphocytes (PBL) response to a dose of γ-rays in patients treated with radioiodine (I-131) for hyperthyroidism vs. healthy controls, to gain information about the individual lymphocytes’ radio-sensitivity. Blood samples were taken from 18 patients and 10 healthy donors. Phosphorylated histone variant H2AX (γ-H2AX) and micronuclei (MN) induction were used to determine the change in PBL radio-sensitivity and the correlations between the two types of damage. The two assays showed large inter-individual variability in PBL background damage and in radio-sensitivity (patients vs. healthy donors). In particular, they showed an increased radio-sensitivity in 36% and 33% of patients, decrease in 36% and 44%, respectively. There was a scarce correlation between the two assays and no dependence on age or gender. A significant association was found between high radio-sensitivity conditions and induced hypothyroidism. PBL radio-sensitivity in the patient group was not significantly affected by treatment with I-131, whereas there were significant changes inter-individually. The association found between clinical response and PBL radio-sensitivity suggests that the latter could be used in view of the development of personalized treatments.

## 1. Introduction

Administration of radioiodine (I-131) is a safe and well-established therapeutic modality for hyperthyroidism, including Graves’ disease (GD), toxic multinodular goiter (TMNG), and toxic adenoma (TA) [1]. Since its introduction in 1941, a huge number of patients have been administered therapeutic activities, and the ease of its administration, lack of significant deterministic adverse, effects, and low cost have prompted widespread use [2,3].

The extensive use of I-131 therapy has raised concerns regarding long-term risks, such as the development of tumors. Still, there is little evidence to support this [4]. The lack of firm epidemiological evidence for late detrimental effects in I-131 treated patients could have several reasons, including the possible onset of an adaptive response (AR). AR is one of the specific cellular responses to low-dose radiation belonging to the group of low-dose phenomena, which also includes the bystander effect (BE), low-dose hyperadiosensitivity/increased radio-resistance (HRS/IRR), and genomic instability (GI). AR refers to the phenomenon where a low-dose ionizing radiation protects the cells from the detrimental effects of a subsequent high dose exposure. Therefore, the AR is the capability of a biological system to respond to a “challenging” dose (cD) when it has been exposed previously to a smaller “priming” dose (pD) of the same or a similar cytotoxic agent [5]. 

Monsieur et al. [6] evaluated the possibility that an AR is triggered in vivo by a protracted exposure, such as that due to the I-131 administration, in patients with thyrotoxicosis or differentiated thyroid cancer, in terms of reduction of MN induction in peripheral blood lymphocytes (PBL) after a gamma-rays cD. However, this population could be non-homogenous in terms of genetic and molecular profiles, and other exposure biomarkers to ionizing radiation could be used, such as phosphorylated histone H2AX variant (γ-H2AX), a marker of DNA double-strand breaks (DSB) [7,8]. 

A study by Schnarr et al. [9] investigated the biological effects of exposure to fluorine 18 fluorodeoxyglucose (F-18-FDG) in terms of AR in the lymphocytes of patients undergoing positron emission tomography (PET) procedures. The endpoints chosen were apoptosis, chromosome aberrations, and γ-H2AX foci induction. The study found an AR for chromosome aberrations, an individual variation in response, and no evidence of adverse cellular effects following a standard F-18-FDG PET scan. 

To our knowledge, there are no other specific studies indicating the presence of an AR in patients exposed to ionizing radiation for medical reasons. Some studies on AR in humans have only been performed ex vivo on lymphocytes taken from healthy donors [10] or refer to cohorts of healthy donors from occupational or environmental exposures [11,12,13,14]. 

Such observations raise the question if there are phenomena capable of modifying the individual’s response to radiation during radiotherapy, resulting either in an adaptation or in a sensitization, and how these modifications can be evaluated at the individual level. 

The present study aimed to assess, in PBL of patients undergoing I-131 therapy for hyperthyroidism and in healthy donors, the existence of either an AR or a sensitization, in terms of H2AX histone phosphorylation, indicative of DNA damage (i.e., early cell damage), and MN induction, indicative of chromosome damage, (i.e., later cell damage) by a gamma-rays challenging dose. This irradiation was used to evaluate the ex-vivo PBL radio-sensitivity, defined as the PBL response to a cD, before or after the I-131 treatment. A detailed analysis of possible correlations between early and late damage has been performed. The entire study has been focused on finding evidence for the inter-individual variability of the response to the I-131 treatment. 

## 2. Results

### 2.1. Radiation-Induced Damage in Terms of γ-H2AX

**Figure 1:**
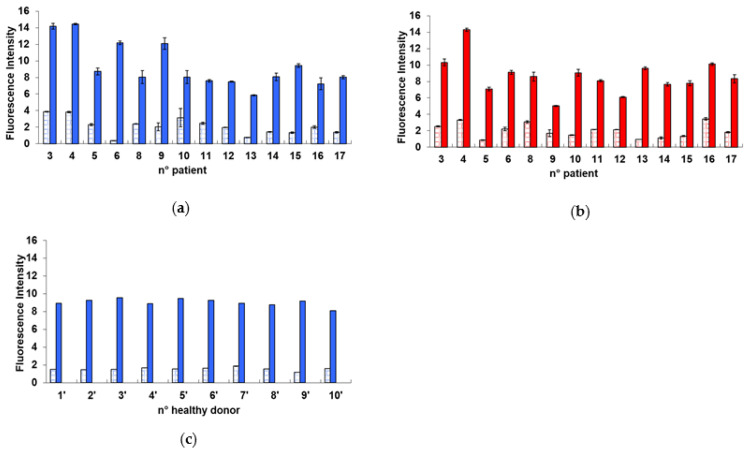
Bar plot with the fluorescence intensity values obtained from PBL tested for H2AX assay. (**a**) Before the I-131 treatment: for each patient, two bars are grouped corresponding to results obtained before and after the cD, respectively (4 Gy). (**b**) After the I-131 treatment: for each patient, two bars are grouped corresponding to results obtained before and after the cD, respectively (4 Gy). (**c**) Healthy donor: the two bars correspond to the results before and after the cD, respectively (4 Gy). The error bars represent the propagation of the errors.

Figure 1 shows the bar plots with the values obtained for each of the 14 patients (panels a and b) and for each of the 10 healthy donors (panel c). It appears that the fluorescence intensity is more uniform for the healthy donors than for the patients, for both the background (i.e., without exposure to the cD) and the response after the cD.

**Figure 2:**
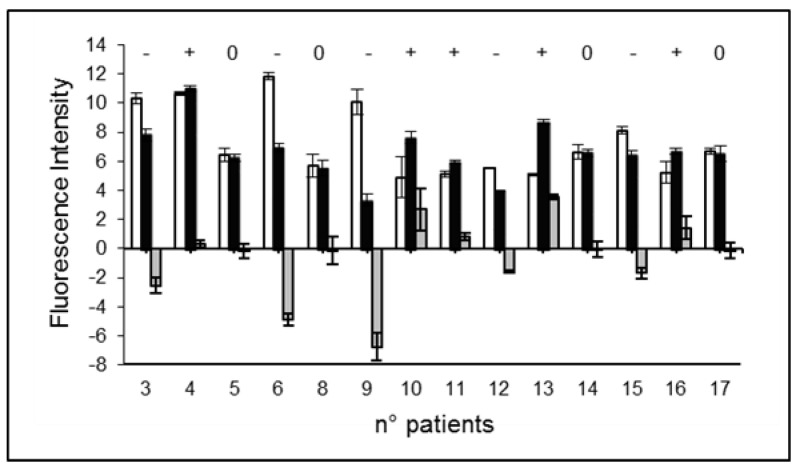
Phosphorylation intensity modulation due to the PBL exposure to the CD (4 Gy) before (white bar) and after (black bar) the I-131 treatment. The third bar represents its difference (delta). Patients marked “+” or “−” showed an increased or decreased responsiveness, respectively, in a statistically significant way; patients marked “0” do not show significant changes. Error bars represent the propagation of the errors.

Figure 2 shows the distribution among the patients of the phosphorylation intensity modulation due to the exposure of PBL to the cD before and after the I-131 treatment and the difference (delta, Δ) between the two values can be assumed as representative of the response modification (i.e., radio-sensitivity or radio-resistance) due to the I-131 treatment. It appears that out of the 14 patients studied, there are 5 with increased responsiveness (36%), 5 with decreased responsiveness (36%), and 4 with no significant changes (28%). Table 1 summarizes the results obtained from the γ-H2AX assay.

### 2.2. Radiation-Induced Damage in Terms of MN Induction

**Figure 3:**
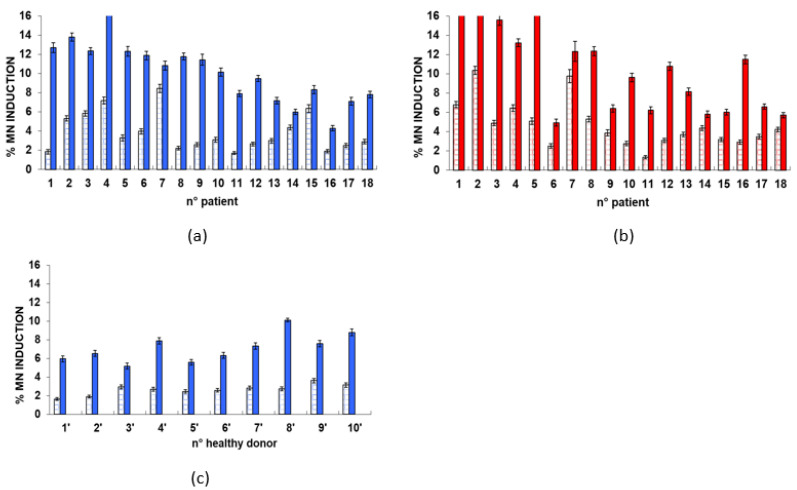
Bar plot for the results of the MN induction. (**a**) Before the I-131 treatment: for each patient, two bars are grouped corresponding to results obtained before and after the cD (1 Gy), respectively. (**b**) After the I-131 treatment: for each patient, two bars are grouped corresponding to results obtained before and after the cD (1 Gy), respectively. (**c**) Healthy donor: the two bars correspond to the results before and after the cD (1 Gy), respectively. The error bars represent the propagation of the error.

Figure 3 shows the bar plots with the values obtained for each of the 18 patients (panels a and b) and each of the 10 healthy donors (panel c). Similarly to the γ-H2AX assay, it appears that the MN frequency is more uniform for the healthy donors than for the patients, both before and after the cD.

**Figure 4:**
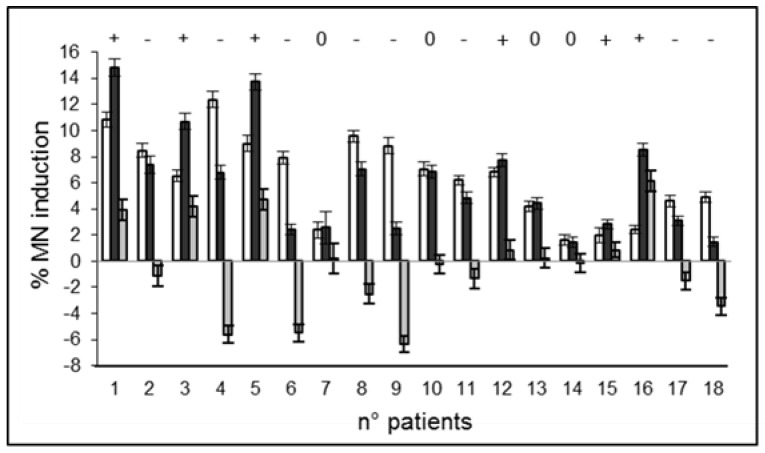
Increase in the MN percentage due to the PBL exposure to the CD (1 Gy) before (white bar) and after (black bar) the I-131 treatment. The third bar represents its difference (delta). Patients marked “+” or “−” showed an increased or decreased responsiveness, respectively, in a statistically significant way; patients marked “0” do not show significant changes. Error bars represent the propagation of the errors.

Figure 4 shows the increase in the MN frequency for each patient due to the exposure of PBL to the cD before and after the I-131 treatment. The third bar for each patient represents the difference (delta, Δ) between these two increases that can be assumed as representative of the response modification due to the I-131 treatment. It appears that among the 18 patients studied, there are 6 with increased responsiveness (33%), 8 with decreased responsiveness (44%), and 4 with no significant changes (22%).

### 2.3. Correlations

The correlations between the two endpoints in terms of background levels, radio-sensitivity, and biological/clinical responses, have been investigated. The correlations in terms of I-131 activity administrated, age, and gender are shown in Supplementary Materials as supplemental data.

- Correlations between background levels and correlations between radio-sensitivity in patients and healthy donors

**Figure 5:**
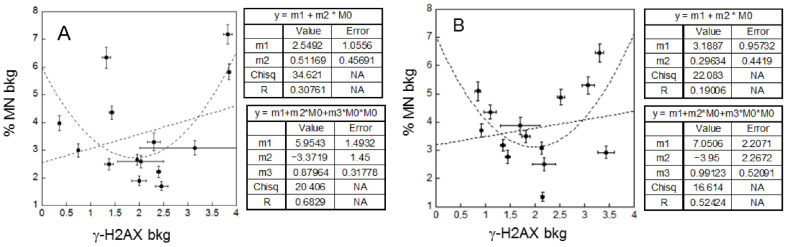
Background MN frequency versus the background level of γ-H2AX in patients, before (**A**) and after (**B**) I-131 treatment. The dotted lines represent linear or parabolic fits of the data. Error bars represent the propagation of the errors. Bkg: background.

The linear fits relative to background levels (i.e., in absence of cD) in patients before and after I-131 treatment give correlation coefficients (R) of 0.31 and 0.19 and errors on the linear coefficients that are bigger or comparable to their values (Figure 5).

These findings indicate that a linear correlation is not very likely in both cases. However, a parabolic fit suits the data much better. In both cases R values are much higher (0.68 and 0.52) than those for the linear fits, indicating the occurrence of a significant correlation. It has to be noted that these data refer to the background damage, i.e., to a damage probably due to endogenous causes and/or to external genotoxic agents such as the natural background radiation. A U-shaped relationship between late and early damage may imply that there is a region at a very low amount of damage where the late damage decreases as early damage increases. 

**Figure 6:**
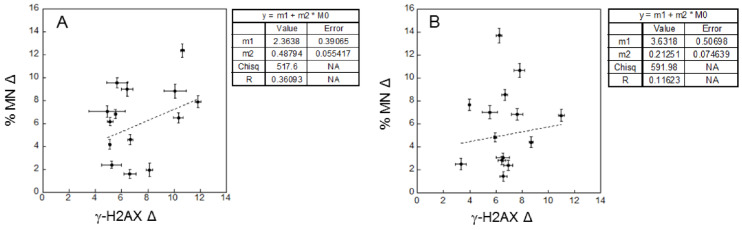
Lymphocytes radio-sensitivity (Delta, Δ) for MN frequency versus γ-H2AX in patients before (**A**) and after (**B**) I-131 treatment. The dotted lines represent linear weighed least-squares fits of the data. Error bars represent the propagation of the errors.

The linear fit relative to radio-sensitivity in patients before and after I-131 treatment gives R of 0.36 and of 0.12, which implies poor correlations (Figure 6). In other words, the radio-sensitivity measured in terms of late damage does not show a significant correlation with that measured by early damage, and this finding holds independently of the I-131 treatment. 

**Figure 7:**
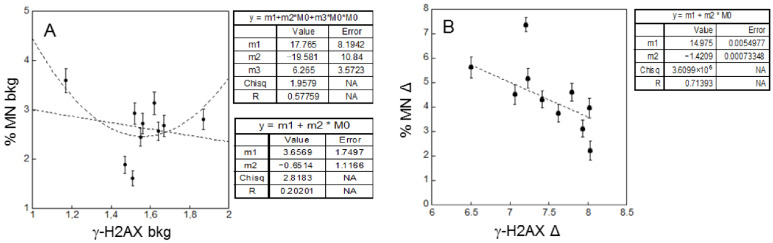
Correlation in healthy donors. (**A**) Background MN frequency versus background level of γ-H2AX. (**B**) Lymphocytes radio-sensitivity (Δ) for MN frequency versus γ-H2AX. The dotted lines represent linear weighed least-squares fits of the data. Error bars represent the propagation of the errors. Background: bkg.

In healthy donors, the results in terms of background (Figure 7, panel A) showed a lack of linear correlation (R = 0.20), similar to that obtained for the patients. On the contrary, we observed a significant linear correlation (R = 0.71) for the radio-sensitivity (Figure 7, panel B) with a negative slope. This means that, after the cD, the increase of MN induction is less for those individuals that have a higher increase of γ-H2AX. This finding is different from that obtained for the patients, where no significant linear correlation is observed.

- Correlation between radiobiological and clinical responses

The possibility that the clinical response, as measured by the outcome at 6 months after the I-131 treatment, is related to radio-sensitivity of the patients, has been explored to get information on whether or not the administered activity is optimal for the patient, depending on his/her radio-sensitivity. Table 2 summarizes, for each patient, the biological response, in terms of radio-sensitivity before the treatment and of radio-sensitivity changes after the treatment, together with the clinical response in terms of the outcome at 6 months. 

To find out if the clinical outcome is statistically related to the radio-sensitivity (both in terms of γ-H2AX and MN induction) before the I-131 treatment, a statistical test has been performed to evaluate the conditional probability of each of the three types of response (hypo-, eu-, hyper-thyroidism) given a certain level of radio-sensitivity before the treatment. To this aim, the patient radio-sensitivity (for each of the two types of assay) has been classified into two categories, i.e., low and high radio-sensitivity, corresponding to responses below or equal to the mean value and above the mean value, respectively (see Table 2). The values for this conditional probability are listed in Table 3. It shows that among the patients showing a high sensitivity (for both γ-H2AX and MN induction), 80% and 82% of them achieved hypothyroidism conditions, respectively. Alternatively, among the patients that achieved hypothyroidism, 82% showed high radio-sensitivity for MN induction. In addition, the majority of patients that achieved euthyroidis showed low radio-sensitivity for both γ-H2AX and MN induction. These data point to an association between high radio-sensitivity before treatment and hypothyroidism response. It can be noted that the achievement of hypothyroidism also occurs in those patients (pts. n. 3, 4, 12, 14) with low administered activity (370–222 MBq), so it appears that the important factor is their radio-sensitivity.

## 3. Discussion

In the present study, the possible modifications of an individual’s response to radiation, consequent to the I-131 therapy, were determined in terms of γ-H2AX and MN induction, established biomarkers of interaction between ionizing radiation and biological systems [15], and reliable biodosimeters in the external-beam radiotherapy [16]. 

γ-H2AX is essentially a marker of DNA double-strand breaks [17] and of early cell damage, widely applied for investigating DNA damage and repair after exposure to ionizing radiation and for evaluating the efficacy of the treatments [18,19]. We quantified γ-H2AX through of flow cytometry as an alternative to scoring γ-H2AX foci by immunofluorescence microscopy. However, the two methods are expected not to be exactly equivalent: although flow cytometry allows fast analysis of thousands of cells, it appears to be less coupled than foci scoring to the number of DNA DSB. It has been proposed that the number of γ-H2AX molecules produced per DSB varies among individuals [20], thus explaining the difference between cytometry and microscopy [21].

The MN induction assay is a marker of chromosome damage such as chromosome breakage and loss after exposure to ionizing radiation [22]. Since it reflects the failure of proper chromosome segregation into daughter cell nuclei, it can be related to unrepaired or misrepaired DNA damage. It has been reported that baseline and induced MN frequencies are determined by genetic factors to a major part [23]. Since there are significant inter-individual differences in the radiation-induced MN, this assay is considered a biomarker of individual radio-sensitivity [24]. In this study, 44% of the patients showed a decreased response in terms of MN induction and 33% increased responsiveness compared to 16% and 1%, respectively, as reported by the study of Monsieur et al. [6]. Moreover, large inter-individual variability was observed in PBL of hyperthyroid patients in terms of both γ-H2AX fluorescence and MN frequency. This larger variability is observed for the background levels and for the response to the cD, and it is not a consequence of the I-131 treatment. It could be related to the different diseases (i.e., to the different genetic and immune status of the patients) submitted to I-131 treatment. GD is an autoimmune disorder in which thyrotropin receptor antibodies (TRAbs) stimulate the TSH receptor, increasing thyroid hormone production [3]. In TA and TMNG, autonomous hormone production can be caused by somatic activating mutations of genes regulating thyroid hormone synthesis [3]. A large inter-individual variability in the dose–response relationship in ionizing radiation-induced focus counts per nucleus in patients with differentiated thyroid carcinoma was reported by Lassmann et al. [25] and by Eberlein et al. [26], by using γ-H2AX and sensor p53-binding protein 1 (53BP1) to the DSB-containing chromatin.

On the contrary, no inter-individual variability in γ-H2AX formation was reported by May et al. [27], who evaluated patients that had undergone PET/Computed Tomography (CT) to differentiate the kinetics of formation and repair of γ-H2AX foci between F-18-FDG and CT-induced DNA lesions. 

From the present results, it is not possible to extract a systematic and consistent dependence of radio-sensitivity in terms of I-131 activity administrated, age, and gender (see Supplementary Material Figures S1–S5). If any, they are masked by the inter-individual differences among patients due to other causes. A trend suggestive of an average decrease with the age in the MN induced by 4 Gy X-rays in a heterogeneous population of donors was observed by Thierens et al. [28], but also in their study, the marked inter-individual differences do not allow firm conclusions. A better insight into the role of age/gender in relation to possible other factors can only be obtained from specific studies with suitable statistical approaches.

Despite the large inter-individual patient variability, the average values of γ-H2AX and MN induction for background levels and the response to the cD is similar to those found in healthy donors, as shown in Table 1. Our results show a poorly significant correlation in the patients between the data on DNA damage and chromosome damage (Figure 5 and Figure 6). This is observed in particular for the radio-sensitivity evaluated by the response to the cD, both before and after the I-131 treatment. As the link between early and late DNA damage is affected by the cell repair machinery, this finding is likely related to the individual variability of repair capability in these patients. It should be noted that part of this variability could be related to the variation among individuals in the number of γ-H2AX produced per DSB [20], as this could affect the fluorescence intensity measured at given DNA damage. As a significant linear (negative) correlation between the radio-sensitivities in terms of early and late DNA damage is found instead for the healthy donors (Figure 7, panel B), it is plausible that the poor correlation found in the patients is related, at least in part, to an additional variability in their response due their pathologic condition.

The U-shaped relationship observed between late and early DNA damage in the background levels of both the patient (Figure 5) and the donor (Figure 7, panel A) groups is noteworthy. Since these groups are not exposed to the cD, this damage can be related to endogenous and/or exogenous agents (the latter include the background radiation and the I-131 treatment) and implies non-linear mechanisms in response to low levels of DNA damage. Indeed, there is mounting evidence of non-linear dose-response to low doses of ionizing radiation [29,30,31], including those typical of the background radiation [32]. This non-linear relation appears to be almost independent, on average, of the patient’s pathological conditions and of the I-131 treatment, even if at an individual level there is no clear indication of a correlation between the change in the background levels of early and late damage caused by the I-131 treatment. 

We now discuss some limitations of the study and their impact merit consideration. The first limitation is the limited number of patients and donors that are considered. Indeed, the complexity and the length of this kind of investigation makes the accrual of a high number of outpatients impractical. Moreover, similar patient sizes are considered in similar studies published in the literature [6,10,25,26]. Increasing the sample size could increase the precision in evaluating the average response and the frequency of subjects deviating from the average. Indeed, for the patients, their number appears enough to show significant deviations in their response, in both directions, from the calculated average value. 

The second limitation is that the patient’s absorbed doses were not calculated, and we were not able to establish a relationship between blood absorbed dose and γ-H2AX and MN induction. Indeed, an evaluation of the individual absorbed dose to the blood would require a radioactive iodine uptake (RAIU) test, whole-body retention measurements, and blood samples at different time intervals after the I-131 administration and then daily during hospitalization [33]. Since all patients were outpatients, this would have required hospitalization, becoming unpractical and a bearer of ethical problems. Several authors suggested applying a fixed dose to treat patients with hyperthyroidism instead of dosimetry-based individual I-131 treatment [34,35]. Fixed activity regimens may ease clinical prescription by eliminating the need for pre-treatment measurement to calculate applied target activity. In a recent metanalysis, there was no significant difference in successful cure of hyperthyroidism between estimated and calculated activities [36]. Although measuring the absorbed dose in the blood is a useful parameter for an appropriate estimation of the I-131 activity to be administered and for a better understanding of the quality of treatment, the success of treatment is not only depending on the administered activity but also on other factors such as thyroid volume, age, and gender of the patient, uptake measurement of iodine, or serum T4 concentration. However, lack of evaluation of the absorbed dose to the blood did not affect the analysis of correlations between early and late DNA damage, because it cannot be expected that the dose is the only quantity determining the biological effects, due to the differences in individual response to radiation.

The results here shown about the correlations between clinical outcome and individual radio-sensitivity support the idea that a customized dose of iodine should be administered to avoid unnecessarily higher radiation exposure.

## 4. Materials and Methods 

### 4.1. Patients and Healthy Donors 

The 18 patients enrolled in this study have been treated with I-131 for hyperthyroidism: 5 with GD, 8 with TA, and 5 with TMNG (Table 4). The study was conducted in accordance with the Declaration of Helsinki, and the protocol was approved by the Institutional Review Board of the Istituto di Medicina Nucleare of the Università Cattolica del S. Cuore. All the patients signed a written informed consent approving their participation in the study. 

The 10 healthy donors have been selected in such a way that they represent a control group matching, as far as practicable, the patient group for what concerns sex and age distributions. To this aim, patients and donors have been grouped into 6 subgroups (3 age subgroups for each gender), as shown in Table 5. 

PBL from patients and healthy donors have been assayed for γ-H2AX and MN induction. In particular, all 18 patients have been tested for MN induction, while only 14 of them have been tested for the γ-H2AX.

### 4.2. Radioiodine Treatment 

The patients were submitted to I-131 treatment: 5 of the 18 patients suffered from GD and received activity of I-131 ranging between 222 and 370 MBq. The others received a fixed activity of I-131 of 600 MBq, administered after withdrawal of thyrostatic drugs (Table 5). All the patients were outpatients, so for practical reasons, neither whole-body retention measurements nor absorbed doses to the blood were performed. After treatment, patients were followed up by clinical assessment and biochemical thyroid function testing, including measurement of levels of TSH, FT3, and FT4, within 1–2 months, at 6 months, and thereafter at 6 monthly intervals. Patients were categorized according to their conditions resulting from clinical and laboratory findings in each follow-up: hyperthyroidism, euthyroidism, and hypothyroidism. Patients with euthyroidism and hypothyroidism were considered cured from the hyperthyroidism.

### 4.3. Lymphocyte Isolation and Culture

**Figure 8:**
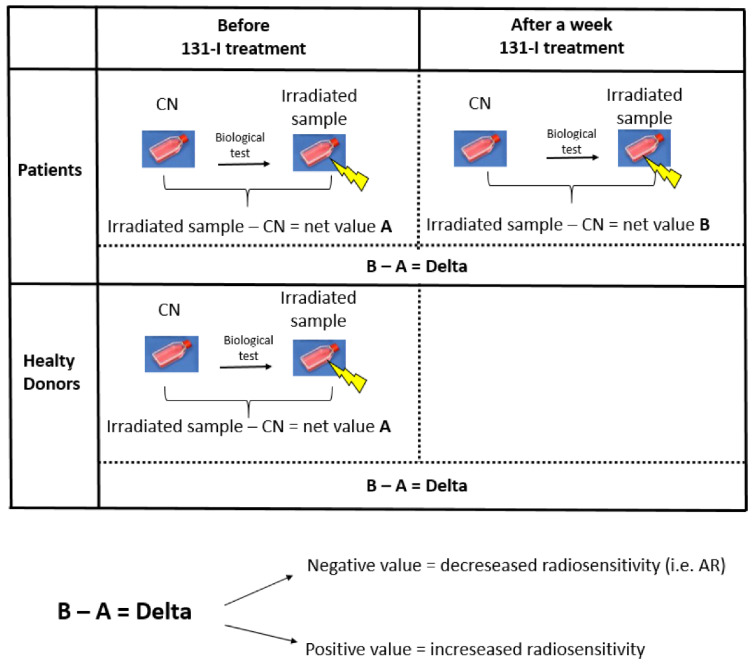
Radiosensitivity’s modulation. The experimental design behind the study and explanation of the delta parameter considered in the results: patients with a negative delta (−) or positive delta (+) show a decreased or increased radio-sensitivity, respectively.

16 mL of blood was collected from each patient before treatment and after 7 days. In the case of healthy donors, blood was collected only once. The samples have been assayed for γ-H2AX and MN induction. Figure 8 shows the experimental design and setting of the study.

For the γ-H2AX assay, 14 mL of the blood was split into two tubes (Vacutainer CPT; BD). The tubes were centrifuged for 30 min at 1800× *g* at room temperature (r.t.). The white coat containing the peripheral blood mononucleate cells (PBMCs) was collected with a Pasteur pipette, transferred to two 15 mL centrifuge tubes (Falcon), and washed twice with Dulbecco’phosphate-buffered saline (DPBS) (GIBCO^®^, Life Technologies, Waltham, Massachusetts, USA) by centrifugation for 15 min at 300× *g* at r.t. Each sample was suspended at an approximate cell concentration of 1 × 10^6^ mL into RPMI 1640 culture medium supplemented with 20% (*v*/*v*) fetal calf serum (FBS), 1% L-glutamine, 1% penicillin/streptomycin (all reagents from GIBCO^®^, Life Technologies), and 0.1% sodium heparin prepared from a stock solution at 50 mg/mL (10 USP/mL, St. Louis, Missouri, USA). Finally, 2% phytohemagglutinin (PHA) prepared from a stock solution at 9 mg/mL (PHA HA15; OXOID S.p.A., Milan, Italy) was added to the medium. The samples were incubated at 37 °C in a humidified atmosphere with 5% CO_2_ for 48 h. 

For MN induction assay, 0.5 mL of fresh blood was added to 4.5 mL of RPMI 1640 medium, supplemented as described above. Four samples were prepared: two were the controls, and two were immediately irradiated. After irradiation, 2% PHA was added to all samples followed by incubation at 37 °C in a humidified atmosphere with 5% CO_2_. 

### 4.4. Irradiation 

The samples prepared for γ-H2AX and MN induction assays were irradiated at r. t. using a ^137^Cs gamma irradiator [Gammacell 40, Nordion Co., Canada] with the doses of 4 Gy and 1 Gy, respectively, and at a dose rate of about 0.9 Gy/min. After irradiation, the samples for the γ-H2AX assay were kept for 2 h at 37 °C in a humidified atmosphere and 5% CO_2_, while the samples for the MN assay were cultured for a further 68 h. This irradiation was used to evaluate the ex-vivo PBL radio-sensitivity defined as the PBL response to a cD, before or after the I-131 treatment.

### 4.5. Flow Cytometry Measurements for γ-H2AX Detection 

The measurement of the amount of γ-H2AX used a protocol modified from Hamasaki et al. [37]. Briefly, 100 μL of PBL fixed in 70% ethanol at r.t and a concentration of 4 × 10^6^ cells/mL was seeded in a 96-well U-bottom plate (NUNC). Cells were washed twice in DPBS, centrifuged at 450 g for 5 min, and resuspended in 100 μL Permeabilization Buffer (PB: 1% BSA [*w*/*v*] and 0.2% Triton X-100 [*v*/*v*] in DPBS). Incubation with 8 μL of mouse monoclonal antiphosphohistone H2AX (Ser139) antibody (Upstate) diluted 1:100 with PB for 30 min at r. t., washing with PB, and then incubation for 20 min at r.t. in the dark with 80 μL of secondary antibody, Alexa 488 F[ab’]2 goat anti-mouse (Molecular Probes) diluted 1:20 with PB. Fluorescence intensity levels of γ-H2AX in 1 × 10^4^ cells were analyzed using LSRII flow cytometry (BD Biosciences). Data analysis was performed by FlowJo software (Tree Star). Radiation-induced γ-H2AX levels were determined as the medians of the fluorescence intensity distributions. The increase in the fluorescent intensity has been calculated for each patient by subtracting the individual background level to provide a more precise evaluation of the lymphocyte radio-sensitivity when applying the cD. 

### 4.6. MN Induction Assay 

MN induction assay was performed according to the protocol described by Fenech [38]. Cytochalasin B (Sigma-Aldrich), at a final concentration of 5.5 μg/mL, was added after 44 h from irradiation. After 24 h, whole blood cultures were centrifuged for 10 min at 250 g at r.t., and resuspended in 5 mL of 0.075 M KCl hypotonic solution for 3 min at r.t. Then, 400 μL methanol:acetic acid (3:5 solution) were added to each sample, and after a second, centrifugation cells were resuspended in 5 mL of ice-cold methanol and kept for 10 min at −20 °C. Then, they were centrifuged and fixed three times with methanol:acetic acid, (7:1 solution) at r.t. The lymphocytes obtained were smeared on the pre-cleaned microscope slides, air-dried, and stored at 4 °C until use. Immediately before the scoring, the coded slides were stained with a 40 μg/mL Acridine Orange (Sigma-Aldrich) solution. Blind scoring of the coded slides was performed using a fluorescence microscope (Leica, Germany) with a 40X il objective. For each sample, 1000 binucleated cells (BNC) with well-preserved cytoplasm were counted in at least four different slides according to the criteria reported by Fenech [34]. The increase in the MN frequency has been calculated for each patient by subtracting the individual background level, which is the same method used for the H2AX phosphorylation assay. 

### 4.7. Data Analysis

Microsoft Excel accomplished the handling of the experimental data and their basic analysis. Graphing and further data analysis were performed using the KaleidaGraph version 3 (Synergy Software, Mt Penn, PA 19606, USA), which also provided the best fit coefficients and correlation coefficients R. 

The correlations were analyzed by plotting the relevant pairs of data for each patient and evaluating the correlation coefficients. Linear least-squared weighted best fits were also calculated for helping inspection of graphics. The correlation probability was evaluated following the criteria reported in Bevington and Robinson’s book [39]. Correlations between radiobiological and clinical responses were analyzed by ad hoc calculations of the conditional probabilities.

## 5. Conclusions

An important finding of this study is that the mean values of the lymphocyte radio-sensitivity, evaluated with both γ-H2AX and MN induction assays when applying the cD, are similar among the patients and the healthy donors, and are not significantly affected by the I-131 treatment. On the contrary, there is a large inter-individual variability that is even enhanced among the patients, possibly due to their pathologic conditions. In the group of patients considered in this study, the number of individuals who, after I-131 treatment, showed increased responsiveness was almost perfectly balanced by the number of those showing reduced responsiveness. These findings, while confirming the conclusions of Monsieurs et al. [6] about the possibility of a sort of AR induced by the I-131 treatment in a significant fraction of the patients, point to the possibility of an increased response in another significant fraction of patients. This is not surprising, given the role played by the inter-individual variability when comparing different groups of patients, and it highlights that the average results cannot be used to make a general prediction on individual patients.

## Figures and Tables

**Table 1:** Results for the γ-H2AX and for the MN induction assays.

	Mean Fluorescence Intensity	Mean Delta Fluorescence Intensity	Mean MN Percentage	Mean Delta MN Percentage
**Patients**				
before I-131, background	2.08 ± 1.03	7.31 ± 2.42	3.84 ± 1.99	6.43 ± 3.17
before I-131, after cD *	9.39 ± 2.71		10.26 ± 3.51	
after I-131, background	1.96 ± 0.87	6.66 ± 1.87	4.67 ± 2.39	6.07 ± 4.01
after I-131, after cD *	8.53 ± 2.23		10.74 ± 5.07	
**Healthy donors**				
no I-131, background	1.56 ± 0.18	7.48 ± 0.49	2.64 ± 0.57	4.48 ± 1.41
no I-131, after cD *	9.04 ± 0.41		7.12 ± 1.53	

* cD = challenging dose.

**Table 2:** Values of radio-sensitivity before the treatment and type of radio-sensitivity changes after the treatment together with the clinical outcome.

Patient No	Radiosensitivity before I−131 (γ−H2AX Assay)	Change in Radiosensitivity (γ−H2AX Assay)	Radiosensitivity before I−131 (MN Assay)	Change in Radiosensitivity (MN Assay)	Outcome at 6 Months
1	NE	NE	10.860	+	Hypo
2	NE	NE	8.4800	−	Hyper
3	10.332	−	6.5100	+	Hypo
4	10.639	+	12.360	−	Hypo
5	6.4320	0	9.0000	+	Eu
6	11.824	−	7.9100	−	Hypo
7	NE	NE	2.3800	0	NE
8	5.6650	0	9.5600	−	Hypo
9	10.069	−	8.8400	−	Hypo
10	4.9050	+	7.0600	0	Hypo
11	5.1320	+	6.1900	−	Hypo
12	5.5300	−	6.8300	0	Hypo
13	5.1010	+	4.1800	0	Eu
14	6.6370	0	1.6200	0	Hypo
15	8.1250	−	1.9700	0	Eu
16	5.2400	+	2.4000	+	Hypo
17	6.6590	0	4.6000	−	Hyper
18	NE	NE	4.9200	−	Eu
Mean value	7.31		5.53		

Positive (+). negative (−) or no (0) changes are evaluated from the difference with respect to the average values. considering significant the differences when exceeding the relevant errors. NE = not evaluated, Hypo = Hypothyroidism, Eu = Euthyroidism, Hyper = Hyperthyroidism.

**Table 3:** Conditional probabilities relating the outcome to the level of radio-sensitivity before the treatment.

Conditional Probability of an Outcome Given a Certain Level of Radio-Sensitivity	γ-H2AX Assay	MN Assay
p(Hypo/low radio-sensitivity)	6/9 (67%)	3/8 (38%)
p(Eu/low radio-sensitivity)	2/9 (22%)	4/8 (50%)
p(Hyper/low radio-sensitivity)	1/9 (11%)	1/8 (13%)
p(Hypo/high radio-sensitivity)	4/5 (80%)	9/11 (82%)
p(Eu/high radio-sensitivity)	1/5 (20%)	1/11 (9%)
p(Hyper/high radio-sensitivity)	0/5 (0%)	1/11 (9%)
**Conditional Probability of a Level of Radio-Sensitivity Given a Certain Outcome**	**γ-H2AX assay**	**MN assay**
p(low radio-sensitivity/Hypo)	6/10 (60%)	2/11 (18%)
p(high radio-sensitivity/Hypo)	4/10 (40%)	9/11 (82%)
p(low radio-sensitivity/Eu)	2/3 (67%)	3/4 (80%)
p(high radio-sensitivity/Eu)	1/3 (33%)	1/4 (25%)
p(low radio-sensitivity/Hyper)	1/1 (100%)	1/2 (50%)
p(high radio-sensitivity/Hyper)	0/1 (0%)	1/2 (50%)
**Conditional Probability of High Radio-Sensitivity from Both Assays Given a Certain Outcome**		
p(Hypo/2xhigh radio-sensitivity)	4/4 (100)%	
p(Eu/2xhigh radio- sensitivity)	0/4 (0%)	
p(Hyper/2xhigh radio-sensitivity)	0/4 (0%)	

**Table 4:** Patient characteristics and administered I-131 activity.

Patient No.	Sex	Age	Pathology	Administered I-131 Activity (MBq)
1	F	43	GD	296
2	M	80	TA	600
3	F	30	GD	370
4	M	62	GD	370
5	F	65	TMNG	600
6	M	71	TMNG	600
7	M	70	TA	600
8	F	66	TA	600
9	M	58	TMNG	600
10	M	78	TA	600
11	F	78	TMNG	600
12	F	69	TMNG	600
13	F	73	TA	600
14	F	59	GD	222
15	M	74	TA	600
16	M	27	GD	222
17	M	47	TA	600
18	M	69	TA	600

GD = Basedow-Graves disease; TA = toxic adenoma; TMNG = toxic multinodular goiter.

**Table 5:** Grouping of patients and healthy donors according to age and gender.

Age Group	Patients (γ-H2AX)			Patients (MN Induction)			Donors (γ-H2AX and MN Induction)		
	M	F	Tot (%)	M	F	Tot (%)	M	F	Tot (%)
≤55	2	1	3 (21%)	2	2	4 (22%)	1	1	2 (20%)
56–69	2	4	6 (43%)	3	4	7 (39%)	2	2	4 (40%)
≥70	3	2	5 (36%)	5	2	7 (39%)	2	2	4 (40%)

M = male; F = female.

## Data Availability

The data presented in this study are available on request from the corresponding author. The data are not publicly available due to limitations of Italian law.

## Supplementary Materials

The supporting information can be downloaded at: https://www.mdpi.com/article/10.3390/ijms231710156/s1.

## Abbreviations

I-131	Radioiodine
γ-H2AX	Phosphorylated histone H2AX variant
MN	Micronuclei
GD	Graves’ disease
TA	Toxic adenoma
TMNG	Toxic multinodular goiter
AR	Adaptive response
BE	Bystander effect
HRS/IRR	Low-dose hyperadiosensitivity/increased radioresistance
GI	Genomic instability
cD	Challenging dose
pD	Priming dose
PBL	Peripheral Blood Lymphocytes
DSB	Double Strand Breaks
DPBS	Dulbecco’phosphate-buffered saline
FBS	Foetal calf serum
PHA	Phytohemagglutinin
F-18-FDG	Fluorine 18 fluorodeoxyglucose
PET	Positron emission tomography
TRAbs	Thyrotropin receptor antibodies
53BP1	p53-binding protein 1
CT	Computed Tomography
