# Supplementary material for: Changes in Radiosensitivity to Gamma-Rays of Lymphocytes from Hyperthyroid Patients Treated with I-131
Source: Int J Mol Sci. 2022 Sep 5;23(17):10156. doi: 10.3390/ijms231710156 (PMC9456272; doi:10.3390/ijms231710156)
Supplement: Supplementary file 1 [file ijms-23-10156-s001.zip › ijms-1846812-supplementary.pdf]

CHANGES IN RADIO-SENSITIVITY TO GAMMA-RAYS OF LYMPHOCYTES FROM HYPERTHYROID PATIENTS TREATED WITH I-131

Supplemental data:

CORRELATIONS

Changes in background levels of damage vs. administered activity

The possibility that the effect of the 131-I treatment affects the background levels of γ-H2AX and MN frequency has been evaluated by plotting this levels against the administered 131-I activity, for each patient. It is worth noting that this activity can significantly differ among the patients depending on their pathology. As mentioned in Materials and Methods the patients with the Basedow-Graves disease received less activity (222-370 MBq) than the others (600 MBq).

Figure S1 shows the changes, after the treatment, in the background levels of γ-H2AX (panel A) and MN frequency (panel B) as a function of the administered activity for each patient. Linear regression lines are obtained by linear least-squares best fits of the data. The correlation coefficient for the γ-H2AX is about 0.5, suggesting a weak positive correlation of the spontaneous frequency of early damage with the administered activity. In contrast, for the MN induction it is less than 0.05, suggesting no significant correlation between the spontaneous frequency of late damage and the administered activity. Figure A1 (panel C) shows that there is no clear indication of a correlation between the change in the background levels of early and late damage caused by 131-I treatment.

Changes in radio-sensitivity vs. administered activity

Figure S2 shows the changes in PBL radio-sensitivity, in terms of γ-H2AX level (panel A) and MN frequency (panel B), after the 131-I treatment as a function of the administered 131-I activity for each patient.

In both cases, the correlation coefficient is very small, and the linear coefficient is not statistically different from zero, suggesting no significant dependency of the PBL radio-sensitivity on the amount of the activity administered to each patient. Panel C shows the correlation between the changes of the two radio-sensitivities. The correlation coefficient of 0.40 indicates a poorly significant correlation between the changes, observed after the 131I treatment, in the radio-sensitivities measured in terms of early or late damage.

Background damage and radio-sensitivity as a function of age and gender in patients

The PBL radio-sensitivity before and after the 131-I treatment as a function of the patient age is shown in Figure S3 and S4, both in terms of γ-H2AX level and MN frequency.

In various cases, the linear fits result in positive or negative slopes, but large errors affected their value. When

the genders are pooled together the age, dependence is significantly different from zero only for the MN radio-sensitivity after the treatment, where a negative slope is observed, mainly due to the female contribution. A significant negative slope is also observed for the age dependence in the female group before the treatment for the radio-sensitivity as measured by the γ-H2AX assay.

On overall, from these observations it is not possible to extract a systematic and consistent dependence of radio-sensitivities on age and/or gender. If any, they are masked by the interindividual differences among patients.

Background damage and radio-sensitivity as a function of age in healthy donors

The PBL radio-sensitivity as a function of the age in healthy donors is shown in Figure S5, both in terms of γ-H2AX level and MN frequency. Due to the limited number of donors, no analysis was performed in terms of gender. The linear fits result, with high statistical significance, in a positive slope for the age dependence for radio-sensitivity as measured by the γ-H2AX assay, and in a negative slope for the MN radio-sensitivity. These trends are more clear-cut than that for patients, since there is much less interindividual variability. An inverse correlation is observed between the radio-sensitivities measured by the two assays.

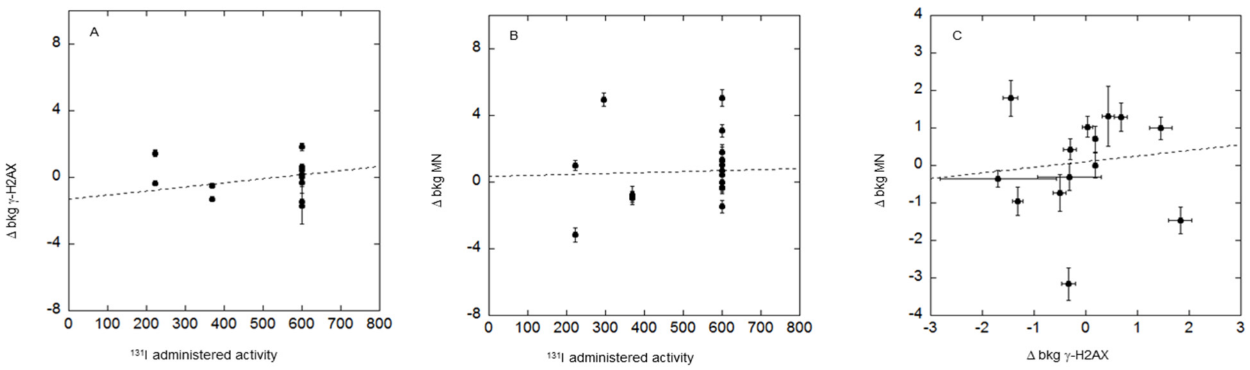

y = m1 + m2 * M0		
	Value	Error
m1	-1,3087	0,13312
m2	0,0024498	0,00022644
Chisq	433,49	NA
R	0,4611	NA

y = m1 + m2 * M0		
	Value	Error
m1	0,34106	0,32683
m2	0,00058986	0,00060143
Chisq	419,85	NA
R	0,047811	NA

y = m1 + m2 * M0		
	Value	Error
m1	0,10095	0,092594
m2	0,15029	0,087317
Chisq	138,42	NA
R	0,14475	NA

Figure S1. Changes in PBL radio-sensitivity (Δ) in the background (bkg) levels of H2AX phosphorylation (A) and MN frequency (B) after the 131-I treatment at various administered activities. Each data point corresponds to one individual patient. (C) Changes in the bkg level of MN frequency plotted against the changes in the bkg level of H2AX phosphorylation. The dotted lines represent linear weighed least-squares fits of the data. The error bars represent the propagation of the error.

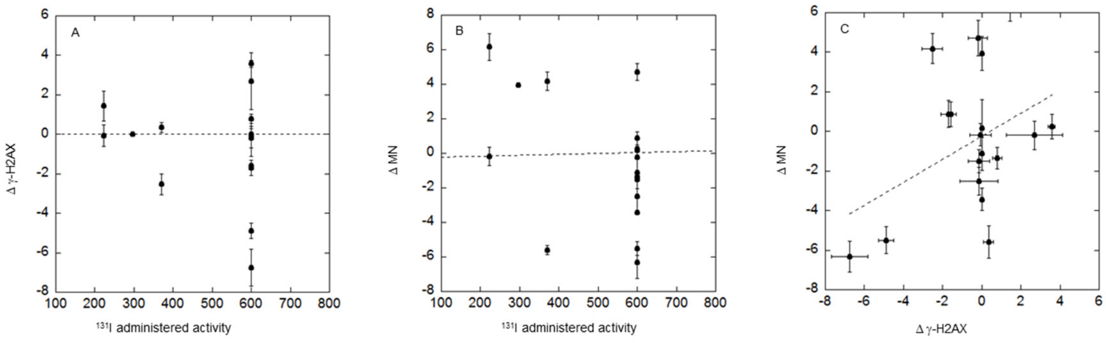

y = m1 + m2 * M0	Value	Error
m1	3,3998e-171	2,0522e-150
m2	6,2385e-174	3,7984e-153
Chisq	14	NA
R	0	NA

y = m1 + m2 * M0	Value	Error
m1	-0,26863	0,26658
m2	0,00052721	0,00052749
Chisq	16,959	NA
R	0,23585	NA

y = m1 + m2 * M0	Value	Error
m1	-0,24845	0,16172
m2	0,5807	0,07017
Chisq	363,74	NA
R	0,39806	NA

Figure S2. Changes in PBL radio-sensitivity (Δ) after the cD in terms of H2AX phosphorylation level (A) and MN frequency (B), after the 131I treatment at various administered activities. (C) shows the relationship between the changes in the two radio-sensitivities. Each data point corresponds to one individual patient. The dotted lines represent linear weighed least-squares fits of the data. The error bars represent the propagation of the error.

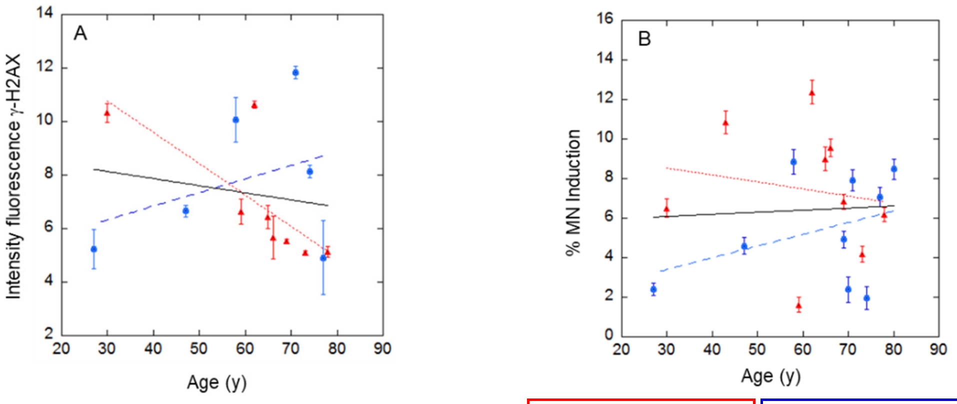

y = m1 + m2 * M0	Value	Error
m1	14,281	2,6843
m2	-0,1171	0,041807
Chisq	15,515	NA
R	0,75275	NA

y = m1 + m2 * M0	Value	Error
m1	4,8126	4,0922
m2	0,050696	0,06646
Chisq	32,897	NA
R	0,35636	NA

y = m1 + m2 * M0	Value	Error
m1	8,9305	2,6854
m2	-0,026562	0,042563
Chisq	73,69	NA
R	0,1773	NA

y = m1 + m2 * M0	Value	Error
m1	9,1734	3,7842
m2	-0,035225	0,058938
Chisq	30,836	NA
R	0,23704	NA

y = m1 + m2 * M0	Value	Error
m1	5,5621	2,3593
m2	0,011656	0,038317
Chisq	10,935	NA
R	0,15037	NA

y = m1 + m2 * M0	Value	Error
m1	7,0405	2,1094
m2	-0,0062668	0,033434
Chisq	45,471	NA
R	0,054029	NA

Figure S3. PBL radio-sensitivity before (A and B) the treatment as a function of the patient age, in terms of H2AX phosphorylation level and MN frequency. Each data point corresponds to one individual patient with gender separation (females = red triangles, males = blue circles). The lines represent linear fits of the data (females = dotted, males = dashed, total = solid lines). The error bars represent the propagation of the error.

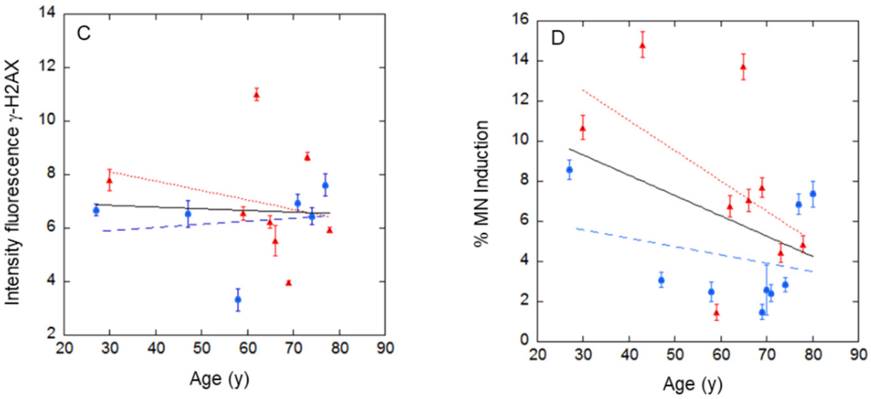

y = m1 + m2 * M0		
	Value	Error
m1	9,5959	5,1569
m2	-0,035326	0,082898
Chisq	87,849	NA
R	0,15902	NA

y = m1 + m2 * M0		
	Value	Error
m1	1,6336	3,745
m2	0,059088	0,057031
Chisq	53,003	NA
R	0,36464	NA

y = m1 + m2 * M0		
	Value	Error
m1	17,056	5,8475
m2	-0,15047	0,093998
Chisq	112,95	NA
R	0,51766	NA

y = m1 + m2 * M0		
	Value	Error
m1	6,8305	3,6797
m2	-0,041492	0,056035
Chisq	51,169	NA
R	0,26951	NA

y = m1 + m2 * M0		
	Value	Error
m1	5,7723	3,217
m2	0,010526	0,050297
Chisq	169,91	NA
R	0,05225	NA

y = m1 + m2 * M0		
	Value	Error
m1	12,34	3,7439
m2	-0,101	0,058535
Chisq	230,13	NA
R	0,39608	NA

Figure S4. PBL radio-sensitivity after (C and D) the treatment as a function of the patient age, in terms of H2AX phosphorylation level and MN frequency. Each data point corresponds to one individual patient with gender separation (females = red triangles, males = blue circles). The lines represent linear fits of the data (females = dotted, males = dashed, total = solid lines). The error bars represent the propagation of the error.

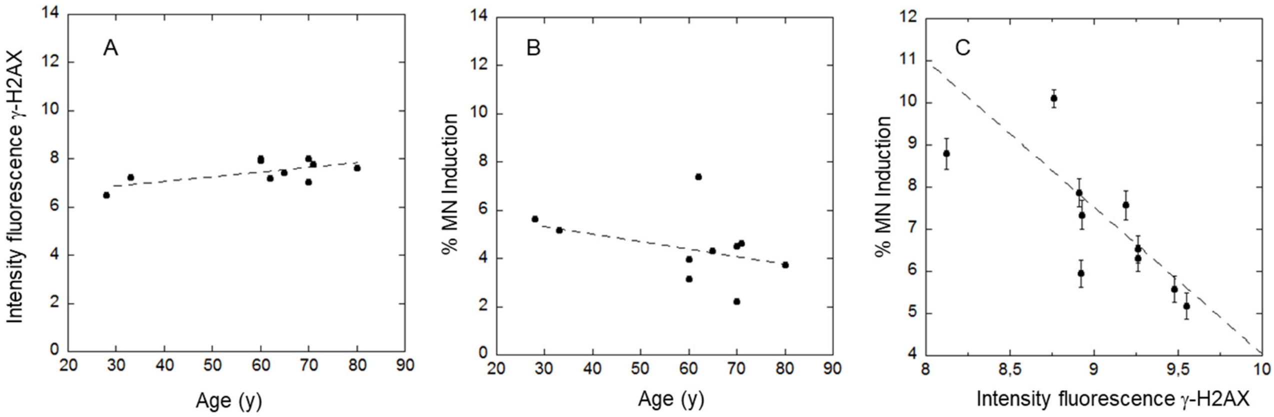

y = m1 + m2 * M0		
	Value	Error
m1	6,2969	0,0014648
m2	0,019283	2,3158e-05
Chisq	1,1654e+06	NA
R	0,61075	NA

y = m1 + m2 * M0		
	Value	Error
m1	38,875	2,3959
m2	-3,4836	0,26501
Chisq	136,31	NA
R	0,74768	NA

y = m1 + m2 * M0		
	Value	Error
m1	6,2541	0,0014648
m2	-0,031086	2,3158e-05
Chisq	5,5605e+06	NA
R	0,49472	NA

Figure S5. PBL radio-sensitivity as a function of the age among the 10 healthy donors, in terms of H2AX phosphorylation level (A) and MN frequency (B). (C) shows the correlation between the PBL radio-sensitivities measured by the two assays. Each data point corresponds to one individual donor. The dotted lines represent linear least-squares fits of the data. The error bars represent the propagation of the error.
